# TP63 as a modulator of ferroptosis in TP53 mutations glioblastoma

**DOI:** 10.1038/s41419-025-07938-w

**Published:** 2025-08-13

**Authors:** Haiping Cai, Jiahao Yang, Feifei Luo, Wu Gan, Yanwen Li, Liang Zhang, Xueying Ke, Alafate Wahafu, Danian Dai, Peng Wang, Dong Zhou

**Affiliations:** 1 Department of Neurosurgery, Guangdong Provincial People’s Hospital (Guangdong Academy of Medical Sciences), Southern Medical University, Guangzhou, China; 2 Guangdong Cardiovascular Institute, Guangdong Provincial People’s Hospital (Guangdong Academy of Medical Sciences), Southern Medical University, Guangzhou, China; 3 Department of Endoscopy, Guangdong Provincial People’s Hospital (Guangdong Academy of Medical Sciences), Southern Medical University, Guangzhou, China; 4 School of Medicine South China University of Technology, Guangzhou, China; 5 Department of plastic surgery, Guangdong Provincial People’s Hospital (Guangdong Academy of Medical Sciences), Southern Medical University, Guangzhou, China

**Keywords:** CNS cancer, Tumour biomarkers

## Abstract

Glioblastoma (GBM) is a highly aggressive brain tumor with limited effective treatment options. Ferroptosis, a form of regulated cell death driven by iron-dependent lipid peroxidation, has emerged as a potential vulnerability in GBM, yet its regulatory mechanisms remain poorly defined. In this study, we investigated the impact of TP53 mutations on ferroptosis sensitivity and identified TP63 as a critical modulator in this process. Integrative transcriptomic and mutational analyses of GBM samples from The Cancer Genome Atlas (TCGA) revealed that TP53 mutations are associated with poor prognosis and altered expression of genes involved in iron homeostasis and glutathione metabolism. Notably, TP63 (mainly ΔNp63 isoform) expression was markedly upregulated in TP53-mutant GBM. Functional experiments demonstrated that TP63 suppresses ferroptosis by reducing reactive oxygen species (ROS) accumulation and lipid peroxidation. Mechanistic studies further showed that TP53 mutations activate the Wnt/β-catenin signaling pathway, leading to nuclear accumulation of β-catenin, which transcriptionally upregulates TP63. In turn, TP63 directly enhances GPX4 expression, a key inhibitor of ferroptosis. These findings define a novel TP53 mutation-Wnt/β-catenin-TP63-GPX4 signaling axis that promotes ferroptosis resistance in GBM and deepen our understanding of ferroptosis regulation in this malignancy.

## Introduction

Glioma accounts for ~30% of intracranial tumors and greater than 70% of malignant brain tumors in adults and has a high mortality and recurrence rate [1]. Grade 4 glioma, also called glioblastoma (GBM), has the worst prognosis among all gliomas [2, 3]. Despite treatment with standard therapy, the median overall survival of GBM is only 14.6–17 months, and the 5-year survival rate is less than 4.7% [4].

TP53, encoding the well-known tumor suppressor protein p53, plays a critical role in maintaining genomic integrity by inducing cell cycle arrest, apoptosis, and other forms of cell death in response to cellular stress. Mutations in TP53 are among the most frequent alterations observed in GBM, contributing to tumor progression and therapeutic resistance, as reported by The Cancer Genome Atlas (TCGA) [5]. Beyond its canonical roles, growing evidence suggests that TP53 also regulates cellular metabolism and contributes to ferroptosis [6, 7], a non-apoptotic form of programmed cell death driven by iron-dependent lipid peroxidation.

Ferroptosis is characterized by the accumulation of lipid-based reactive oxygen species (ROS) [8], depletion of intracellular glutathione (GSH), and reduced activity of glutathione peroxidase 4 (GPX4), which normally detoxifies lipid peroxides [9]. When GPX4 function is impaired, excess lipid peroxides and iron-mediated Fenton reactions trigger oxidative damage, culminating in ferroptotic cell death [10–14]. Recent studies have shown that TP53 can promote ferroptosis by inhibiting the expression of SLC7A11, a key component of the cystine/glutamate antiporter system Xc−, thereby depleting intracellular cystine and reducing GSH synthesis [15, 16]. TP53 can also delay ferroptosis through activation of CDKN1A/P21, a cyclin-dependent kinase inhibitor that promotes cell cycle arrest and limits oxidative stress under certain conditions [17].

The dual role of TP53 in ferroptosis appears to depend on cellular context, metabolic state, and the nature of TP53 mutations. While wild-type (WT) TP53 may sensitize cells to ferroptosis under oxidative stress or cystine deprivation [18], mutant TP53 often loses this regulatory function or may acquire new oncogenic properties that promote tumor survival. However, the precise mechanisms by which TP53 mutations influence ferroptosis pathways in GBM remain poorly understood.

In this study, we investigated the ferroptosis regulatory network associated with TP53 mutations in GBM using bioinformatics and experimental approaches. We identified TP63, a member of the p53 gene family, as a downstream effector that is upregulated in TP53-mutant GBM and contributes to ferroptosis resistance. Mechanistically, TP53 mutations activate the Wnt/β-catenin signaling pathway, leading to increased stabilization and nuclear accumulation of β-catenin. Elevated β-catenin transcriptionally upregulates TP63, which in turn promotes GPX4 expression and suppresses lipid peroxidation and ROS accumulation, thereby facilitating glioma cell survival and resistance to ferroptotic cell death. These findings suggest that TP63 may play a key role in mediating ferroptosis escape in TP53-mutant GBM and could serve as a potential molecular target for therapeutic intervention or biomarker development in this patient population.

## Result

### TP53 mutations are frequently observed in GBM and induce metabolic changes

**Fig. 1:**
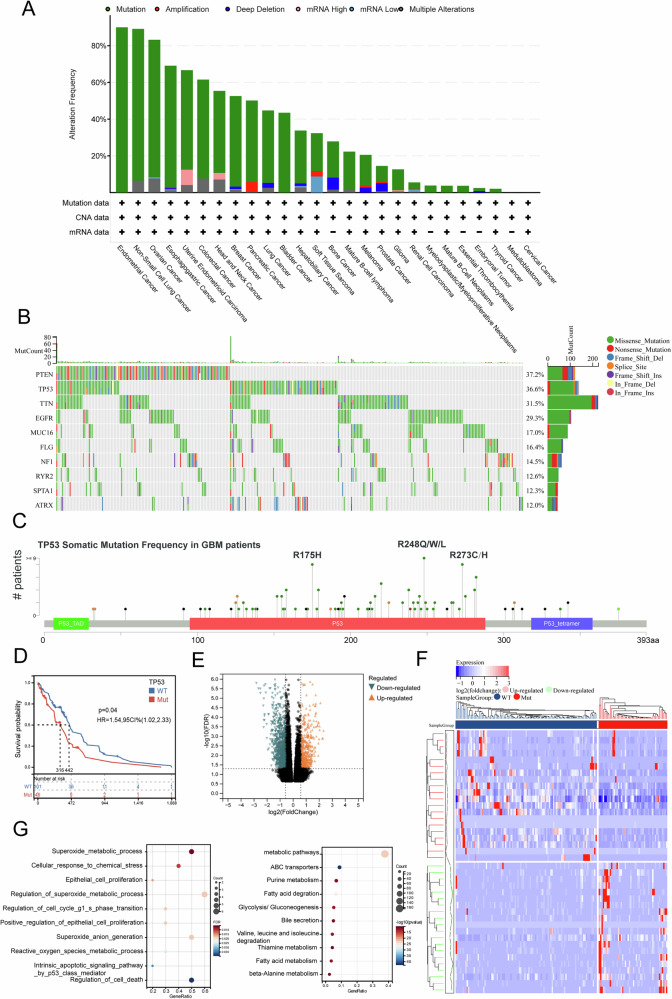
TP53 mutations are frequently observed in GBM and induce metabolic changes. **A** Pan-cancer analysis of mutation frequencies from the cbioportal database (https://www.cbioportal.org/). **B** Waterfall plot showing the top 10 mutated genes in the TCGA-GBM cohort. **C** Common somatic mutation sites of TP53 in GBM patients. **D** Kaplan–Meier survival analysis of GBM patients stratified by TP53 mutation status using the TCGA database. **E**, **F** Differential gene expression analysis of TP53-mutant and TP53 wild-type patients in the TCGA-GBM cohort. **G** Enrichment analysis of differentially expressed genes between TP53-mutated samples and TP53 wild-type samples. *p < 0.05, **p < 0.01, ***p < 0.001, ****p < 0.0001.

We first examined the mutation frequency of TP53 across various cancers using data from cbioportal database, which revealed that TP53 mutations are prevalent in multiple tumor types (Fig. 1A). Similarly, mutation frequency analysis in patients with GBM also showed a high prevalence of TP53 mutations (Fig. 1B). Furthermore, the somatic mutation sites of TP53 in GBM patients were similar to those observed in most tumors, clustering around R175H, R248Q/W/L, and R273C/H (Fig. 1C). Next, Kaplan–Meier survival analysis based on TP53 mutation status indicated that GBM patients with TP53 mutations had significantly poorer prognosis than those with WT TP53 (Fig. 1D). To investigate the mechanisms by which TP53 mutations influence GBM, we performed differential expression genes (DEGs) analysis on transcriptomic data from patients with TP53 mutations and those with WT TP53 (Fig. 1E, F). Enrichment analysis of the differentially expressed genes revealed a concentration in pathways related to oxygen and lipid metabolism (Fig. 1G). Given that previous studies have shown a close relationship between oxygen and lipid metabolism and ferroptosis, these findings suggest a potential link between TP53 mutations and ferroptosis in GBM patients.

### TP53 mutations inhibit ferroptosis

**Fig. 2:**
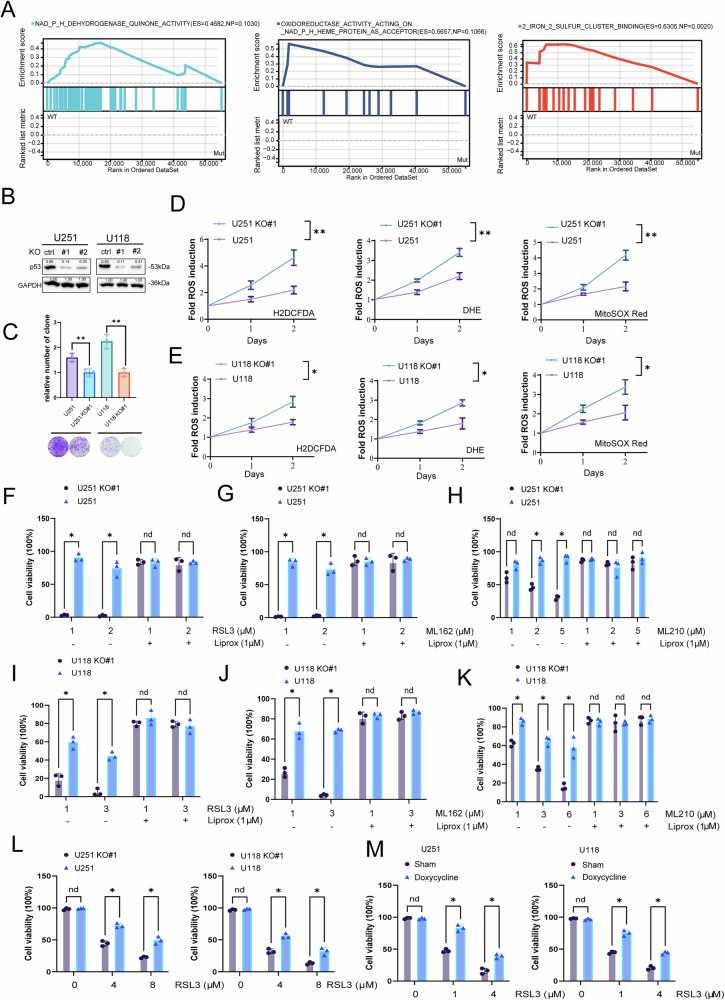
TP53 mutations inhibit ferroptosis. **A** Gene Set Enrichment Analysis (GSEA) of differentially expressed genes between TP53-mutant and wild-type samples. **B** Western blot analysis of p53 in the indicated cells. **C** Comparison of colony formation between TP53 knockout and TP53-mutant cell lines. **D**, **E** Flow cytometric analysis of intracellular ROS levels in TP53-mutant and TP53 KO glioma cells at different time points (days). **F**–**K** Cell viability assays of TP53-mutant and TP53 KO cells under ferroptosis-inducing conditions (RSL4/ML162/ML210). **L** Cell viability assays of TP53-KO and untreated cells at high cell density in the presence of RSL3. **M** The viability of U251(p53 R273H) and U118(p53 R213Q) tumor cells, which were infected with a doxycycline-inducible lentivirus expressing sgRNAs targeting TP53, was assessed after treatment with either sham or doxycycline (1 μg/ml) for 48 h and then treated with RSL3 for an additional 24 h. (*n* = 3 biological replicates per group, ns not significant, *p < 0.05, **p < 0.01, ***p < 0.001, ****p < 0.0001).

To investigate the potential impact of TP53 mutations on glioblastoma (GBM) patients, we conducted gene set enrichment analysis (GSEA) using transcriptomic data. The results revealed that GBM tumors with WT TP53 exhibited significantly higher enrichment scores (ES) for pathways related to redox processes and iron-sulfur cluster binding (Fig. 2A). These findings suggest that TP53-mutant GBM displays enhanced antioxidant capacity compared to WT TP53 GBM, thereby indicating a potential suppression of ferroptosis in tumors harboring TP53 mutations. To further investigate the role of TP53 mutations in regulating ferroptosis in GBM, we selected the U251 (TP53 R273H) and U118 (TP53 R213Q) glioblastoma cell lines, both of which harbor homozygous TP53 mutations. Using CRISPR-Cas9 gene editing, we generated TP53 knockout cell lines, referred to as U251 KO and U118 KO (Fig. 2B). Additionally, we reintroduced the TP53 R273H mutant via plasmid overexpression into the U251 KO and U118 KO cells (Supplementary Fig. 1A). In colony formation assays, the TP53-mutant U251 and U118 cells exhibited significantly enhanced clonogenic potential compared to their respective TP53 knockout counterparts. (Fig. 2C). To further investigate the regulatory role of TP53 mutations on ferroptosis, we used three different fluorescent probes, H2DCFDA, DHE, and MitoSOX Red to detect intracellular reactive oxygen species (ROS). The results showed that the TP53-mutant U251 and U118 cells had lower ROS levels compared to U251 KO and U118 KO, indicating that ferroptosis was inhibited in these cells (Fig. 2D, E). To directly assess the impact of mutant p53 on ferroptosis, we treated U251 and U251 KO cells with RSL3, a GPX4 inhibitor known to induce ferroptosis. Compared to the TP53-mutant U251 cells, U251 KO cells exhibited significantly increased sensitivity to RSL3-induced cell death. Co-treatment with Liproxstatin-1, a ferroptosis inhibitor, fully rescued the cells, confirming that loss of mutant p53 sensitizes cells to ferroptosis (Fig. 2F). Consistent with these findings, U251 KO cells also showed heightened sensitivity to two additional GPX4 inhibitors, ML162 and ML210, relative to the TP53-mutant parental line. Liproxstatin-1 again effectively reversed the cytotoxic effects, confirming ferroptosis as the mode of cell death (Fig. 2G, H). We next examined the response of U118 (TP53 R213Q) cells and their TP53-knockout counterpart, U118 KO, to RSL3. Similar to U251 cells, the U118 KO cells were markedly more sensitive to RSL3, and this effect was again reversed by Liproxstatin-1 (Fig. 2I). Increased sensitivity to ML162 and ML210 was also observed in U118 KO cells, with Liproxstatin-1 fully rescuing cell viability (Fig. 2J, K). Importantly, even under high cell density conditions, known to confer resistance to ferroptosis, TP53-deficient cells (U251 KO and U118 KO remained significantly more vulnerable to RSL3-induced death than their TP53-mutant counterparts (Fig. 2L). To further validate the role of mutant p53, we employed a doxycycline-inducible CRISPR-Cas9 system to acutely delete TP53 in U251 and U118 cells using a lentiviral vector expressing a TP53-targeting sgRNA (Supplementary Fig. 1B). Upon doxycycline induction, cells exhibited markedly increased sensitivity to RSL3-induced ferroptosis compared to vehicle-treated controls (Fig. 2M). Collectively, these results strongly support the conclusion that mutant p53 protects glioma cells from ferroptosis by regulating GPX4 activity. TP53-deficient clones were consistently sensitive to GPX4 inhibitors (RSL3, ML210, and ML162), while remaining unresponsive to erastin, which induces ferroptosis through a GPX4-independent mechanism (Supplementary Fig. 1C). Overall, our findings demonstrate that deletion of mutant p53 enhances ferroptotic sensitivity, an effect that is effectively reversed by Liproxstatin-1.

### TP53 mutations activate Wnt/β-catenin signaling in GBM

**Fig. 3:**
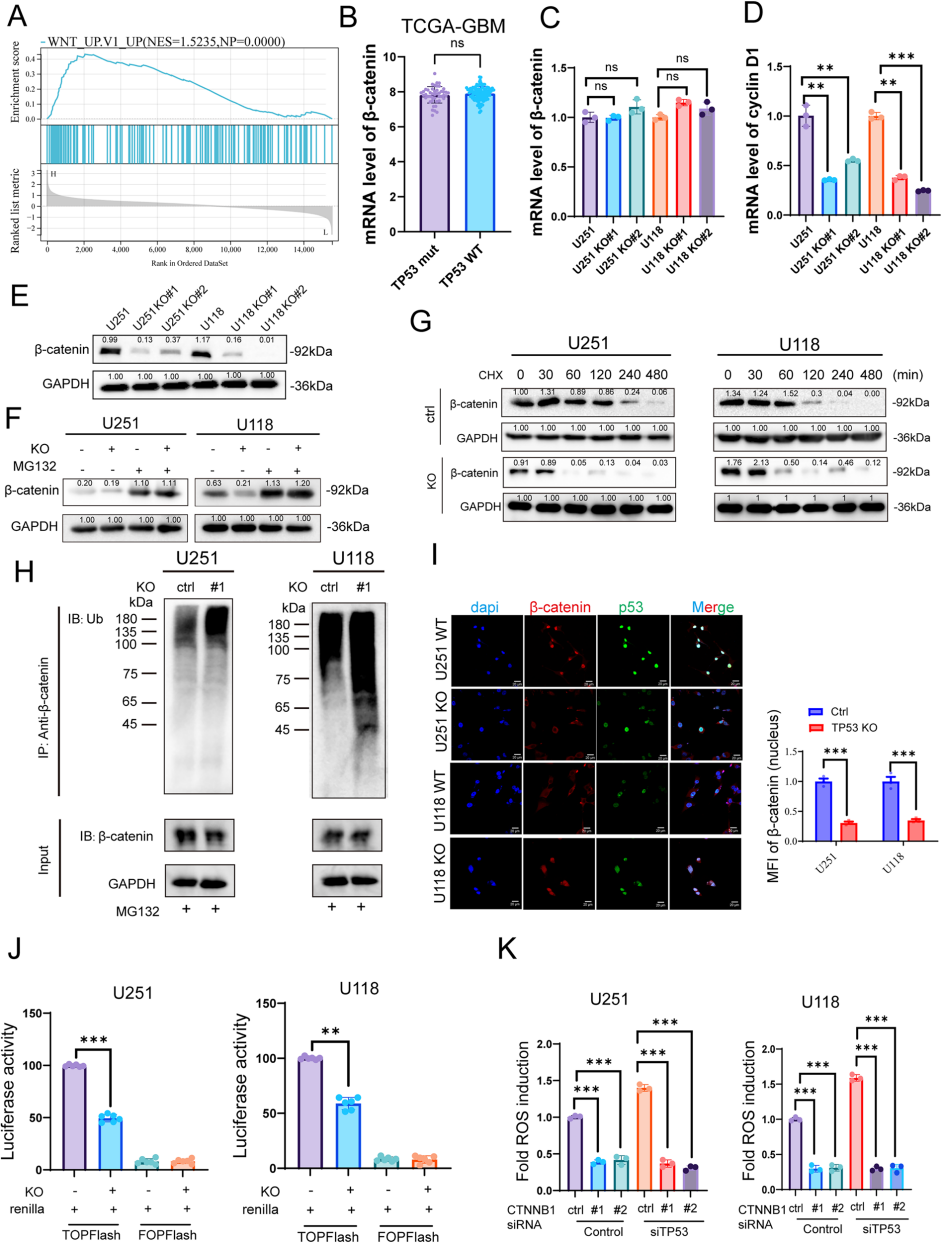
TP53 mutations activate Wnt/β-catenin signaling in GBM. **A** Gene Set Enrichment Analysis showing enrichment of the Wnt pathway in TP53 mutation-mediated genes. **B** mRNA levels of β-catenin in TP53-mutant and wild-type samples from TCGA. **C**, **D** qPCR analysis of β-catenin and cyclin D1 mRNA expression levels after TP53 KO. **E** Western blot analysis of β-catenin protein levels after TP53 KO. **F** Western blot analysis of β-catenin protein levels in TP53 KO and untreated groups with or without MG132 treatment for 24 h. **G** Western blot analysis of β-catenin protein levels in TP53 knockout and untreated groups with cycloheximide (CHX) treatment in different times. **H** Immunoprecipitation (IP) of β-catenin followed by immunoblotting (IB) with anti-ubiquitin (Ub) antibody in U251 and U118 wild-type (ctrl) and TP53 knockout (KO) cells. Cells were treated with MG132 to inhibit proteasomal degradation. Increased ubiquitination of β-catenin was observed in KO cells compared to wild-type controls. **I** Representative immunofluorescence images of β-catenin (red), p53 (green), and nuclei (DAPI, blue) in U251 and U118 wild-type and TP53-KO cells. Merged images show co-localization. Scale bar, 20 μm. Right panel: Quantification of mean fluorescence intensity (MFI) of nuclear β-catenin. Data are shown as mean ± SEM (*n* = 3); **J** β-catenin activity following mutant p53 knockout as measured by the TOPFlash luciferase reporter system (TLRS). **K** The effect of β-catenin knockdown on glioma cell ROS induction with or without TP53 knockdown. (*n* = 3 biological replicates per group; ns not significant, *p < 0.05, **p < 0.01, ***p < 0.001, ****p < 0.0001).

Subsequently, GSEA following differential gene expression analysis revealed that TP53 mutation-mediated genes were enriched in Wnt pathway (NES = 1.5235, NP = 0.0000) (Fig. 3A), suggesting that TP53 mutation may regulate Wnt/β-catenin signaling pathway. We then detected mRNA of β-catenin (CTNNB1) in the TP53 mutation GBM patients and TP53 WT GBM patients from TCGA. Results showed that there was no significant difference in β-catenin mRNA levels between two groups, as well as between the TP53 mutation group and the TP53 KO group in cell lines. However, the mRNA level of cyclin D1, decreased after TP53 KO (Fig. 3B–D). At the protein level, we found that β-catenin decreased with TP53 KO (Fig. 3E). Therefore, we speculate that TP53 mutation may participate in the Wnt/β-catenin pathway by regulating β-catenin protein levels and this effect might be attributable to proteasomal degradation because decreased β-catenin levels were recovered by the proteasomal inhibitor MG-132 (Fig. 3F). Furthermore, in cycloheximide (CHX) treatment assay, TP53 KO in glioma cells decreased β-catenin stabilization, shortening the half-life of β-catenin (Fig. 3G). To investigate the mechanism by which mutant p53 regulates β-catenin stability, we performed immunoprecipitation followed by immunoblotting using an anti-ubiquitination antibody. Following treatment with the proteasome inhibitor MG132, TP53 KO cells displayed markedly increased polyubiquitination of β-catenin compared to their p53-mutant counterparts in both U251 and U118 cell lines (Fig. 3H), suggesting that mutant p53 stabilizes β-catenin by preventing its ubiquitin-mediated degradation. We further evaluated β-catenin nuclear localization using immunofluorescence staining. In both U251 and U118 TP53 KO cells, nuclear β-catenin levels were significantly reduced compared to WT controls (Fig. 3I), as quantified by mean fluorescence intensity (MFI) in the nucleus. To more precisely evaluate the effect of TP53 on Wnt/β-catenin signaling activity, we employed a dual-luciferase reporter assay using TOPFlash and FOPFlash constructs co-transfected with Renilla luciferase as an internal control. TOPFlash activity reflects β-catenin-mediated transcriptional activation of the Wnt pathway, whereas FOPFlash serves as a negative control for nonspecific transcriptional activity. Following normalization to Renilla luciferase, we observed significantly reduced TOPFlash activity in TP53 KO U251 and U118 cells compared to their WT counterparts, indicating that loss of mutant p53 impairs Wnt/β-catenin signaling (Fig. 3J). To investigate whether β-catenin contributes to the ferroptosis phenotype in p53 mutation tumors, we performed an experiment designed to rescue the diminished ROS induction following β-catenin and TP53 knockdown. After siRNA-mediated knockdown of β-catenin, TP53 knockdown could not restore ROS induction in U251 and U118 cells, underscoring the essential role of β-catenin downstream of mutant p53 (Fig. 3K). Overall, these findings indicate that mutant p53 promotes β-catenin nuclear accumulation and may enhance β-catenin transcriptional activity in GBM cells.

### Wnt/β-catenin signaling upregulates TP63 expression in TP53-mutant GBM

**Fig. 4:**
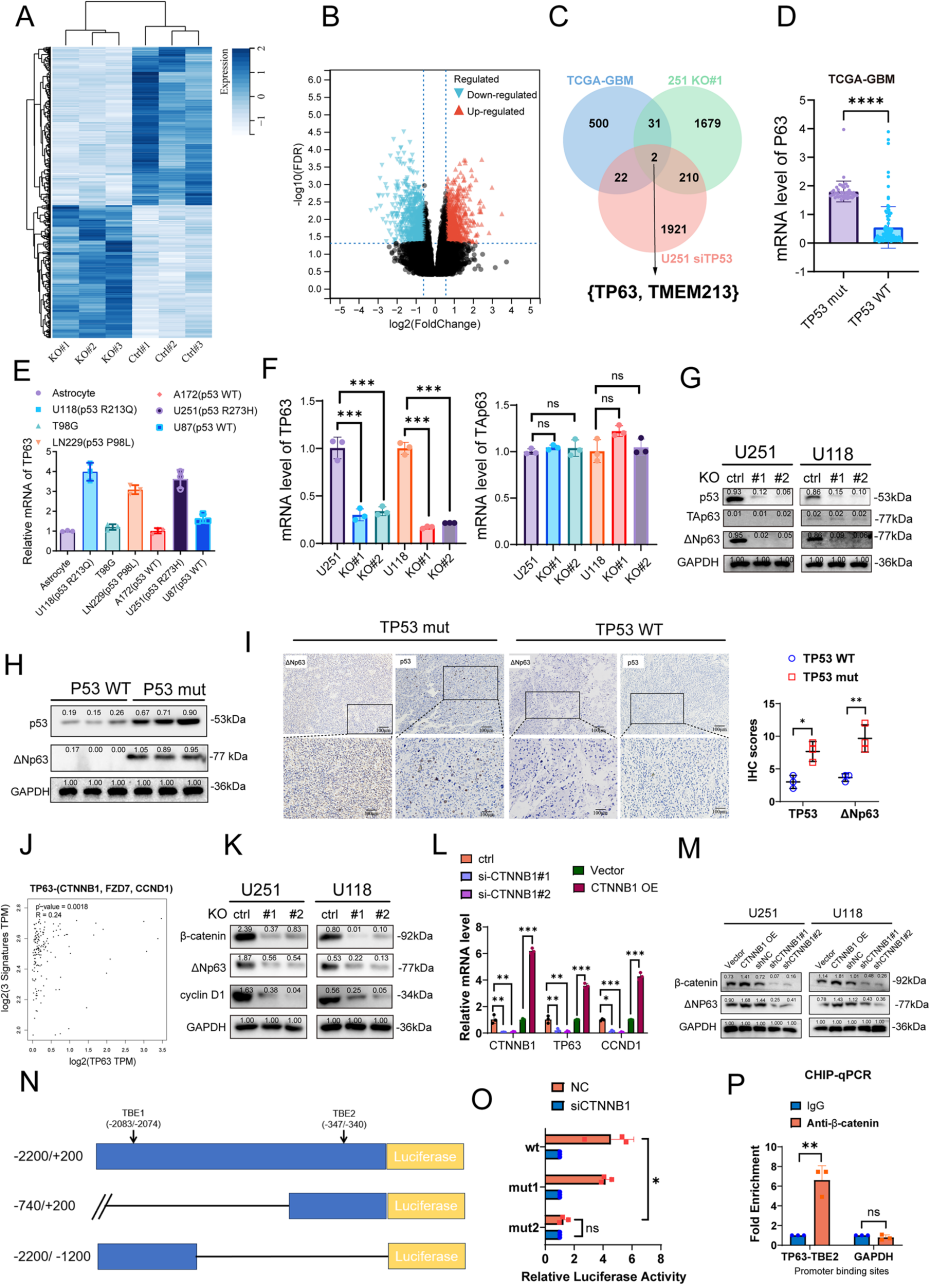
Wnt/β-catenin signaling upregulates TP63 expression in TP53-mutant GBM. **A** Heatmap comparing transcriptome sequencing data from glioma cell lines (U251(p53 R273H) vs. U251(KO)). **B** Volcano plot analysis the differential genes between U251(p53 R273H) and U251(KO) groups. **C** Venn diagram showing the intersection of genes changes among three cohorts: GBM patients with or without TP53 mutation from the TCGA database; U251(KO) and U251(p53 R273H) cell lines; U251(p53 R273H) and U251(siTP53) derived from the GEO database (GSE35454). **D** Comparison of p63 mRNA expression levels between TP53 wild-type and TP53 mutation groups in the TCGA-GBM cohort. **E** TP63 mRNA expression levels in different cell types. **F**, **G**. mRNA and protein level of TAp63 and ΔNp63 expression after TP53 KO, analyzed by qPCR and Western blot. **H**, **I** Immunohistochemical staining and western blot analysis shows higher ΔNp63 expression in TP53-mutated patients. Scar bar = 100 μm. **J** Correlation analysis of TP63 with Wnt signaling pathway-related genes (β-catenin (CTNNB1), cyclin D1 (CCND1), Frizzled-7 (FZD7)) using the GEPIA2 database. **K** Western blot analysis of β-catenin, ΔNp63 and cyclin D1 protein levels after TP53 knockout. **L** qRT-PCR analysis of CTNNB1, TP63, and CCND1 mRNA levels in U251 cells transfected with two independent siRNAs targeting CTNNB1 (siCTNNB1#1 and #2) or CTNNB1 overexpression plasmid. **M** Western blot showing reduced or increased β-catenin and ΔNp63 protein levels following CTNNB1 knockdown or overexpression in U251 and U118 cells. **N** Schematic representation of luciferase reporter constructs driven by the TP63 promoter (−2200 to +200) with two predicted TCF/LEF sites (TBE1 and TBE2), along with single mutant versions (mut1: TBE1-mutated; mut2: TBE2 mutated). **O** Relative luciferase activity of TP63 promoter constructs in U251 cells with or without CTNNB1 knockdown. Mutation of TBE2 abolished β-catenin-mediated promoter activation. **P** ChIP-qPCR analysis using anti-β-catenin antibody showing enrichment at the TP63 promoter (TP63-TBE2) compared to IgG control; GAPDH served as a negative control locus. (*n* = 3 biological replicates per group; ns, not significant, *p < 0.05, **p < 0.01, ***p < 0.001, ****p < 0.0001).

To investigate the mechanisms by which TP53 mutations promote GBM malignancy, we performed transcriptome sequencing analysis on total RNA extracted from U251 (Ctrl) and U251 (KO) cells (Fig. 4A). GSEA following differential gene expression analysis revealed that TP53 mutation-mediated genes were enriched in glutathione metabolism (NES = 1.3884, NP = 0.0000), suggesting that TP53 mutation may regulate ferroptosis through glutathione metabolic activity (Fig. 4B and supplementary Fig. 2A). We next analyzed TP53 mutation-associated DEGs in GBM patients from the TCGA database; in U251(KO) and U251(Ctrl) cell lines; in U251(Ctrl) and U251(siTP53) derived from the GEO database. The overlap of DEGs among these three groups was visualized using a Venn diagram, revealing two common DEGs: P63 and TMEM213 (Fig. 4C). Using the mRNA data of GBM patients from the TCGA database, we confirmed that TP63 mRNA levels were higher in TP53-mutant patients than in TP53 WT patients (Fig. 4D). Furthermore, TP63 mRNA levels were higher in cell lines with TP53 mutations (Fig. 4E). Since TP63 has two major isoforms, TAp63 and ΔNp63, we measured the mRNA levels of total TP63 and TAp63 separately using isoform-specific primers. The results showed that TP53 knockout did not affect TAp63 expression but led to a significant decrease in total TP63 mRNA levels, suggesting that the ΔNp63 isoform may be selectively regulated by mutant TP53. This finding was further validated at the protein level by western blot analysis using isoform-specific antibodies against TAp63 and ΔNp63. Notably, TAp63 expression was low or undetectable in both U251 and U118 glioma cells, suggesting that ΔNp63 is the predominant isoform in these cell lines and may play a more relevant role in glioma biology. (Fig. 4F, G). We also confirmed on clinical samples that ΔNp63 expression levels were higher in GBM patients with TP53 mutations (Fig. 4H, I). In addition, we used immunofluorescence staining (IF) to observe ΔNp63 expression after TP53 KO, which showed that ΔNp63 expression was also reduced following TP53 KO (Supplementary Fig. 2B). Next, we investigated whether ΔNp63 expression had an impact on the prognosis of GBM patients. Kaplan–Meier survival analysis of all GBM patients using the GEIPIA2 database showed no significant difference in survival based on TP63 expression (p = 0.18). while on TP53-mutant GBM patients, patient with higher TP63 expression was associated with poor prognosis ((Supplementary Fig. 2C). Furthermore, in the GEPIA2 database, we included Wnt pathway-related genes (β-catenin, cyclin D1 (CCND1), Frizzled-7 (FZD7)) for correlation analysis with TP63, and the results demonstrated that TP63 is involved in the Wnt pathway (R = 0.24, Fig. 4J). β-catenin, ΔNp63 and cyclin D1 decreased with TP53 KO (Fig. 4K). To explore whether β-catenin directly regulates TP63 expression, we silenced CTNNB1 (β-catenin) in GBM cells using two independent siRNAs. qRT-PCR analysis showed that knockdown of CTNNB1 significantly reduced the mRNA levels of TP63 and CCND1, as expected (Fig. 4L). Consistently, western blot analysis revealed that β-catenin knockdown markedly decreased ΔNp63 protein levels in both U251 and U118 cells (Fig. 4M), indicating that β-catenin positively regulates ΔNp63 expression. We next constructed a series of TP63 promoter luciferase reporter plasmids to functionally validate the β-catenin-responsive elements. The full-length TP63 promoter (−2200 to +200) containing two predicted TCF/LEF sites (TBE1 and TBE2) showed robust luciferase activity that was significantly suppressed upon CTNNB1 knockdown (Fig. 4N, O). Mutation of TBE2 completely abolished β-catenin responsiveness, confirming that β-catenin directly activates TP63 transcription via this cis-elements. To further determine whether β-catenin directly binds to the TP63 promoter, we performed chromatin immunoprecipitation (ChIP)-qPCR using an anti-β-catenin antibody. Significant enrichment of β-catenin was detected at TBE2 site of the TP63 transcription start site (Fig. 4P), supporting direct transcriptional regulation. Overall, these results provide compelling evidence that Wnt/β-catenin signaling upregulates ΔNp63 expression in TP53-mutant GBM.

### TP63 promotes GPX4 expression and suppresses ferroptosis in GBM cells

**Fig. 5:**
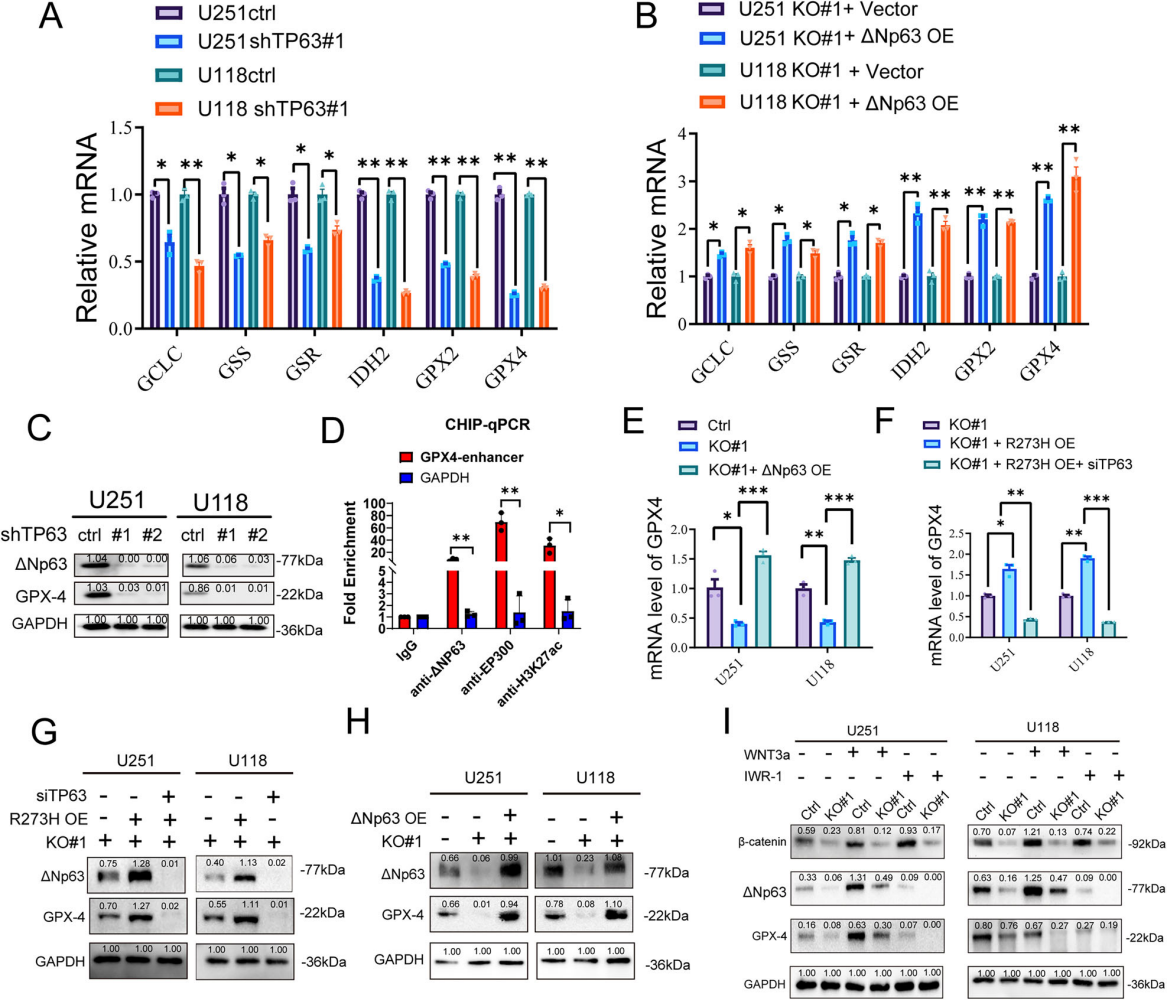
TP63 promotes GPX4 expression and suppresses ferroptosis in GBM cells. **A, B** qPCR analysis of the mRNA of ferroptosis-related genes (GCLC, GSS, GSR, IDH2, GPX2, GPX4) in different TP63 level glioma cells. **C** Western blot analysis of ΔNp63 and GPX4 protein levels in U251 and U118 cells transduced with two independent shRNAs targeting TP63 (shTP63#1 and #2) or control shRNA (ctrl). GAPDH served as the loading control. Densitometric quantification is shown below each band. **D** ChIP-qPCR analysis of TP63 (ΔNp63), transcriptional coactivator EP300, and the active histone mark H3K27ac at the GPX4 enhancer region in U251 cells. IgG and GAPDH promoter served as negative controls. **E**–**H** qPCR and Western blot analyses examining the effect of TP63 knockdown or overexpression on GPX4 expression in TP53-mutant or KO conditions. **I** Protein levels of β-catenin, ΔNp63, and GPX4 in TP53 KO and control groups under WNT3A (150 ng/ul) or IWR-1 (1 μM) treatment for 24 h. (*n* = 3 biological replicates per group; ns, not significant, *p < 0.05, **p < 0.01, ***p < 0.001, ****p < 0.0001).

To investigate the role of TP63 in regulating ferroptosis, we examined the mRNA levels of key enzyme genes associated with ferroptosis (GCLC, GSS, GSR, IDH2, GPX2, GPX4) after TP63 knockdown or overexpression. The results showed that p63 knockdown or overexpression downregulated or upregulated the expression levels of these enzymes, respectively (Fig. 5A, B). GPX4 is a major regulator of ferroptosis, and GPX4 knockout leads to the accumulation of reactive oxygen radicals on membrane lipids, inducing ferroptosis. Next, we aimed to investigate whether TP53 regulates GPX4 via ΔNp63. We first assessed GPX4 protein levels following TP63 knockdown in U251 and U118 GBM cell lines. Western blot analysis revealed that silencing TP63 markedly reduced GPX4 expression in both cell lines (Fig. 5C), suggesting a potential transcriptional regulatory relationship. We next performed ChIP-qPCR to evaluate the binding of ΔNp63 to the enhancer region of the GPX4 gene. Notably, ChIP assays showed strong enrichment of ΔNp63 at the GPX4 enhancer region compared with the GAPDH negative control locus. In addition, enrichment of p300, a transcriptional co-activator, and H3K27ac, a histone modification associated with active enhancers, was also observed at the same locus (Fig. 5D). These results support that ΔNp63 directly binds to the GPX4 enhancer region and cooperates with transcriptional coactivators to promote GPX4 transcription in GBM cells. We further showed that TP53 KO led to a reduction in GPX4 expression, an effect that was reversed by p63 overexpression. On the other hand, in U251 KO and U118 KO glioma cells, TP53 R273H mutation resulted in an upregulation of GPX4, which was reversed by siTP63 treatment (Fig. 5E–H). These results confirmed that TP53 modulates GPX4 expression through ΔNp63. Finally, we explored the regulatory mechanism by which TP53 influences GBM sensitivity to ferroptosis. To examine whether the interactions between TP53, TP63, and GPX4 depend on β-catenin signaling, we used Wnt3A stimulation and the β-catenin inhibitor IWR-1. Wnt3A treatment induced ΔNp63 and GPX4 expression in control cells but not in TP53 KO cells, while IWR-1 treatment inhibited the expression of both ΔNp63 and GPX4 (Fig. 5I). In conclusion, our data suggest that TP63 promotes GPX4 expression and suppresses ferroptosis in GBM cells.

### TP63 mediates ferroptosis resistance and glioma malignancy induced by TP53 mutations

**Fig. 6:**
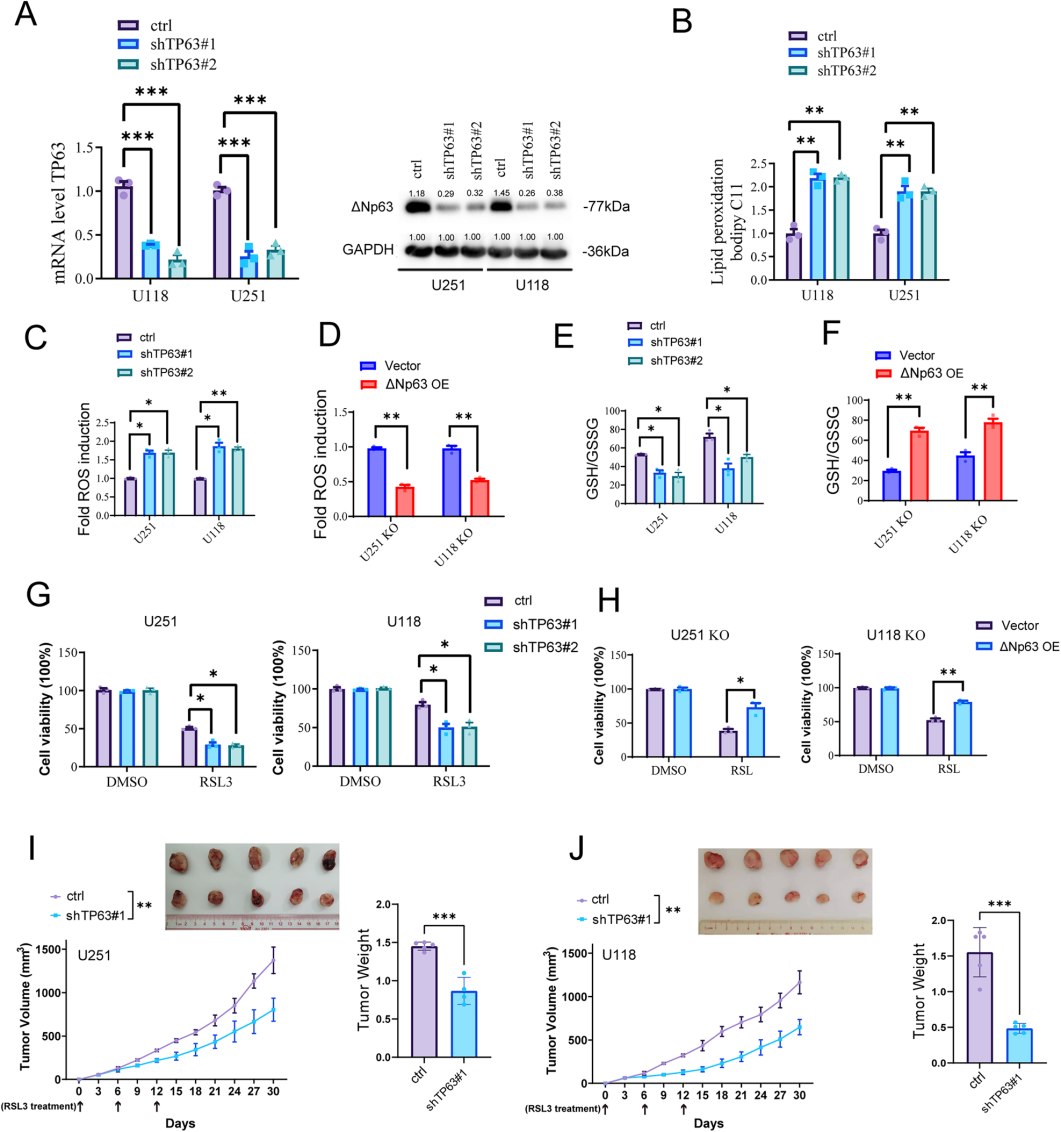
TP63 mediates ferroptosis resistance and glioma malignancy induced by TP53 mutations. **A** qPCR and Western blot showing the efficacy of shRNA-mediated p63 knockdown. **B**–**D** Flow cytometric analysis of lipid peroxidation and ROS levels in cells with different p63 levels. **E**, **F** Analysis of GSH/GSSG levels in cells with different p63 levels. **G**, **H** Measuring the cell activity of tumor cells with different p63 levels and treat with or without RSL3. **I** Tumor cells with or without p63 knockdown were injected into nude mice (*n* = 5). The growth rates of tumors derived from these cells were monitored every 3 days following their injection into nude mice, with intratumoral administration of RSL3 at the specified time points (arrows). (*n* = 3 biological replicates per group; ns not significant, *p < 0.05, **p < 0.01, ***p < 0.001, ****p < 0.0001).

Next, we sought to determine whether TP53 mutation regulates ferroptosis sensitivity and glioma malignancy in tumor cells through ΔNp63. We first downregulated ΔNp63 expression using two siRNAs targeting TP63 mRNA (Fig. 6A) and detect lipid peroxidation and ROS levels used flow cytometry. The results showed that compared to the control group, tumor cells exhibited increased lipid peroxidation and ROS levels after TP63 was silenced (Fig. 6B, C), indicating that TP63 silencing promoted ferroptosis in tumor cells. Conversely, overexpression of p63 in U251KO and U118 KO cells inhibited ferroptosis (Fig. 6D). The ratio of glutathione to glutathione disulfide (GSH/GSSG) can be used to assess cellular redox status and evaluate the cellular ferroptosis state. Our results demonstrated that the GSH/GSSG ratio decreased following the downregulation of ΔNp63 expression (Fig. 6E). In contrast, the opposite effect was observed in U251 KO and U118 KO cells upon ΔNp63 overexpression (Fig. 6F). Subsequently, we treated tumor cells with RSL3 and found that TP63 knockdown increased the sensitivity of the cells to ferroptosis, while TP63 overexpression reduced their sensitivity to ferroptosis (Fig. 6G, H). To evaluate the functional role of ΔNp63 in vivo, we generated U251 and U118 tumor cell lines with stable TP63 knockdown (shTP63) or a control vector (ctrl) and established subcutaneous tumor formation mouse models. Tumor growth was monitored every 3 days, and we observed slower tumor progression in the shTP63 group compared to the ctrl group, suggesting that TP63 knockdown sensitized tumors to RSL3 treatment (Fig. 6I, J). In conclusion, our results demonstrate that TP63 mediates ferroptosis resistance and glioma malignancy induced by TP53 mutations.

## Discussion

**Fig. 7:**
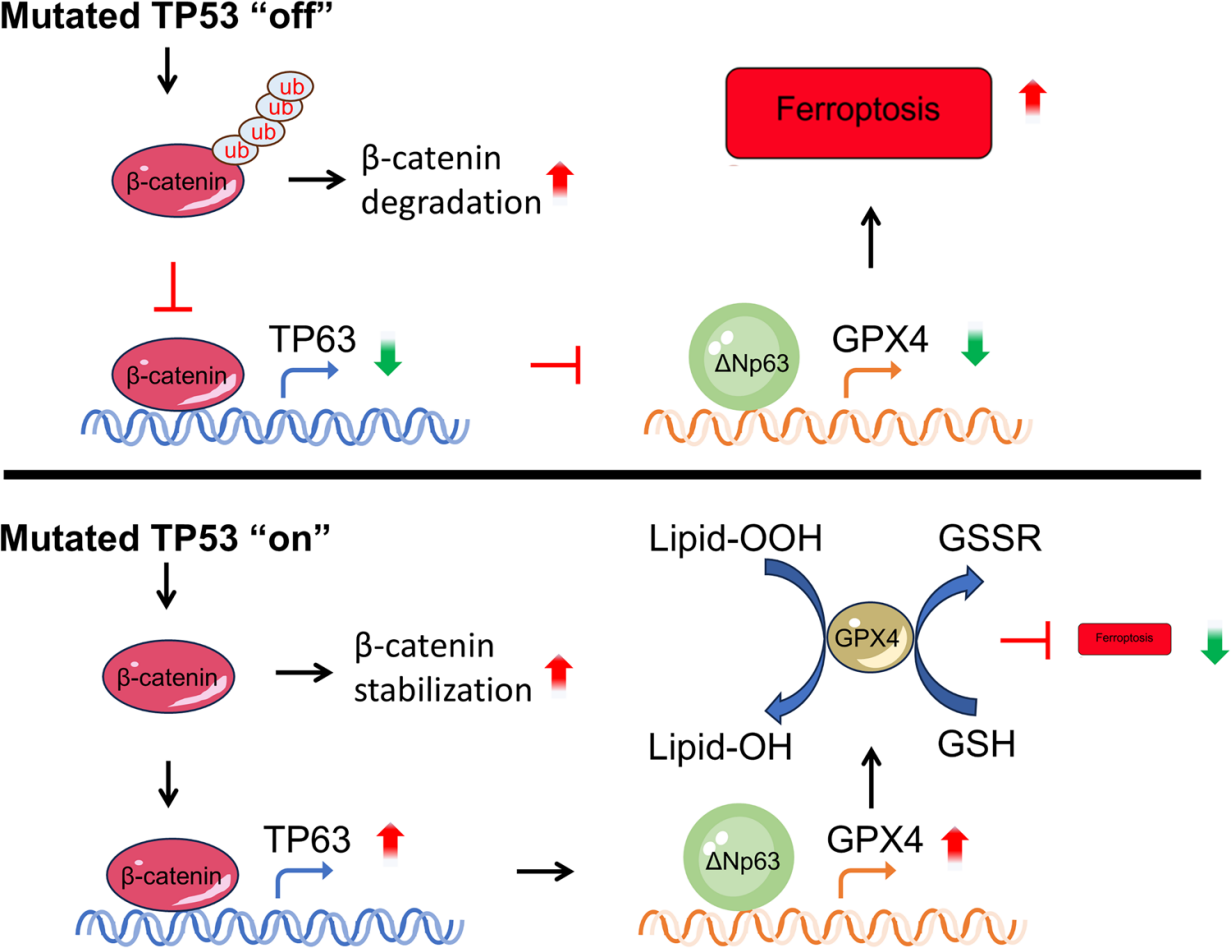
Schematic model of TP53 mutation-mediated ferroptosis resistance via Wnt/β-catenin-TP63-GPX4 axis in glioblastoma. TP53 mutations activate Wnt/β-catenin signaling by reducing ubiquitination of β-catenin, leading to nuclear accumulation of β-catenin, which transcriptionally upregulates TP63 expression. Predominantly, ΔNp63 promotes GPX4 expression by binding to its enhancer region and driving transcription. Elevated GPX4 expression suppresses lipid peroxidation and confers ferroptosis resistance, ultimately promoting GBM cell survival and malignancy.

In this study, we demonstrated that TP53 mutations are prevalent in GBM and that TP53 mutations inhibit lipid peroxidation and reduce ROS levels in tumor cells, leading to the suppression of ferroptosis. We further demonstrated that TP53 mutations regulate ferroptosis by activating the Wnt/β-catenin pathway to upregulate ΔNp63, subsequently promoting GPX4 expression (Fig. 7). This study contributes to a better understanding of the pathogenesis and potential therapeutic interventions for TP53-mutant GBM.

TP53 is a tumor suppressor gene that plays a crucial role in various key cellular processes, including cell cycle regulation, DNA repair, and apoptosis [19]. TP53 mutations are found in approximately half of human cancers and are typically associated with poor prognosis. Studies on GBM patients and cells have shown that TP53 mutations confer resistance to therapy [20]. Therefore, developing new therapeutic strategies targeting the TP53 mutant phenotype is of utmost importance. Previous studies have indicated that TP53 is a major regulator of metabolism, controlling glucose, lipid, amino acid, nucleotide, iron and redox processes [21]. It suppresses anabolic pathways (such as de novo lipogenesis and nucleotide synthesis) while promoting catabolic pathways, including oxidative phosphorylation, lipolysis and fatty acid oxidation. Ferroptosis is an iron-dependent, regulated form of cell death that has gained significant attention for its potential role in cancer therapy. In this study, we analyzed public datasets and found that TP53 mutations are highly prevalent in GBM patients and are associated with poor prognosis. GSEA analysis further revealed that TP53 mutations are closely linked to oxygen and lipid metabolism. Functional assays demonstrated that TP53 mutations inhibit lipid peroxidation and ROS levels, indicating that TP53 mutations promote cancer progression by regulating ferroptosis. Furthermore, through transcriptomic sequencing and analysis of public datasets, we identified p63 as a downstream target that is activated by TP53 mutations through the Wnt/β-catenin signaling pathway, contributing to ferroptosis regulation.

Wnt/β-catenin is an evolutionarily conserved pathway that plays a critical role in cell growth and differentiation. β-catenin is a multifunctional protein that serves as an important component of adherens junctions and a key transcriptional activator in the Wnt pathway. Studies have shown that activation of Wnt/β-catenin signaling is associated with various cancers. For example, inactivation of APC leads to β-catenin accumulation and overactivation of Wnt target genes, promoting colorectal cancer development [22]. GSEA analysis revealed that TP53 mutation-mediated genes were enriched in Wnt pathway. Subsequently, we found that p63 is particularly associated with Wnt/β-catenin signaling, which we later validated in molecular biology experiments. Although previous studies have established that Wnt/β-catenin signaling is involved in GBM progression, the relationship between TP53 mutation and Wnt/β-catenin signaling has not been well explored. Our experimental results showed that TP53 knockdown in glioma cells did not affect the mRNA levels of β-catenin, but it did reduce β-catenin protein levels. This suggests that TP53 mutations may impact β-catenin protein levels through post-transcriptional regulation. To further investigate, we performed MG-132, CHX treatment and IP assays, which confirmed that TP53 mutations influence the protein stability of β-catenin. Based on these findings, we hypothesize that TP53 mutations activate the Wnt/β-catenin signaling pathway, leading to the upregulation of ΔNp63 expression and the inhibition of ferroptosis. To test this hypothesis, we performed that ChIP-qPCR and found that β-catenin directly binds to the TP63 promoter.

In conclusion, this study demonstrates that TP53 mutations, which are frequently observed in GBM, contribute to ferroptosis resistance and tumor progression through a defined molecular pathway. By integrating transcriptomic data and performing functional validations, we reveal that TP53 mutations activate the Wnt/β-catenin signaling pathway, which in turn upregulates the expression of ΔNp63, a dominant TP63 isoform. ΔNp63 directly enhances the transcription of GPX4, a key ferroptosis suppressor, thereby reducing lipid peroxidation and promoting GBM cell survival. These findings uncover a TP53 mutation-β-catenin-ΔNp63-GPX4 axis that drives ferroptosis resistance and glioma malignancy, highlighting ΔNp63 as a potential therapeutic target in TP53-mutant GBM.

## Materials and methods

### Data acquisition and differential expression gene (DEG) analysis

Patient data on relative clinical pathological parameters (Age, Gender, KPS, overall survival etc). were acquired from the public database TCGA (http://cancergenome.nih.gov/). The R package limma was then utilized for exploring the differentially expressed genes in these datasets. The differential expression of individual genes was determined by log2(fold change) (log2FC) and adjusted p-value. A total of 148 GBM patients with complete clinical data were enrolled in our cohort for further study. The mRNA expression profiles of U251 glioma cells with or without siTP53 treatment were acquired from GEO database (GSE35454) (https://www.ncbi.nlm.nih.gov/geo/). Tumor experiments were conducted in accordance with the guidelines and approved by the Ethics Committee of Guangdong Provincial People’s Hospital (KY-N-2022-018).

### Enrichment analysis

The potential functions of DEGs were analyzed by GO and KEGG enrichment analysis using the R software cluster profiler package (version 3.14.3). The underlying biological mechanism of TP53 mutation in GBM samples was evaluated by GSEA using the R package clusterProfiler (version 3.14.3).

### Plasmid transduction

pcDNA3.1(+)-ΔNp63 were purchased from Obio company and transiently transfected into glioma cells using Lipofectamine® 3000 and p3000 (Invitrogen, Carlsbad, CA, USA). For genes knockdown, siRNAs targeting TP63 or β-catenin were purchased from RiboBio Technology (Guangzhou, Guangdong, China). For genes knockout, A pool of two sgRNA plasmids targeting human TP53 was purchased from iGeneBio (Guangzhou, China) and performed according to the manufacturer’s instruction.

### Immunofluorescence

The protein level of ΔNp63 was determined in U251 and U118 glioma cells with or without TP53 KO using immunofluorescence assay. Cells were seeded at a density of 5000 cells/well on confocal dishes. After adherence, cells were washed twice with PBS and fixed with 4% formaldehyde (freshly prepared, pH 7.4) for 10–20 min at room temperature or 4 °C. Cells were washed three times with PBS for 5 min each, and then permeabilized with 0.1–0.5% Triton X-100 in PBS for 5–10 min at room temperature. Cells were blocked with goat serum for 30–60 min at room temperature. Cells were washed three times with PBS for 5 min each. The cells were then incubated with the primary antibody overnight at 4 °C. The next day, cells were washed three times with PBS for 5 min each. Cells were then incubated with a fluorescent secondary antibody for 1 h at room temperature in the dark, followed by three washes with PBS for 5 min each. After incubation with DAPI for 10 min at room temperature in the dark, cells were observed using a fluorescence microscope.

### Immunohistochemistry

The expression level of ΔNp63 and p53 protein in glioma tissues were detected by immunohistochemical staining. Simply, sections were probed with primary antibodies overnight at 4 °C, and the antibodies were detected using the DAB system (Golden Bridge, Beijing, China). The used antibody were listed in Supplementary table 2.

### Western blot analysis

Western blot analysis was performed following the standard protocol. Briefly, protein extracts were prepared by lysing cells or tissues in RIPA buffer containing protease and phosphatase inhibitor cocktails (MedChem Express). Protein concentrations were quantified with a BCA assay kit (Thermo Scientific). Equal concentrations of protein (30 μg) were separated and transferred onto polyvinylidene difluoride membranes (EMD Millipore). The membranes were then probed with the appropriate primary antibody followed by HRP-conjugated secondary antibodies. The used antibody were listed in Supplementary Table 2.

### Animal models

Targeting human TP63 were inserted into the pLKD-CMV-G&PR-U6-shRNA vector and were used to establish U251shTP63-ctrl, U251shTP63#1, U118shTP63-ctrl and U118shTP63#1 cell lines (OBiO Technology, Shanghai, China). Four-week-old female Balb/c-nu/nu mice were purchased from Guangdong medical laboratory animal center (Guangdong, China). Twenty mice were random allocated into four groups (U251shTP63-ctrl; U251shTP63#1; U118shTP63-ctrl; U118shTP63#1) and implanted with 2.5 × 10^5^ tumor cell to establish Subcutaneous xenograft monitored every 3 days. When the tumors grew to a size of about 6 to 7 mm in diameter, they were all treated with intratumoral injections of RSL3 (100 mg/kg) on days 0, 6, and 12 Tumor sizes were calculated using the following formula: tumor volume = 0.5 × length × (width)^2^. Five weeks later, the mice were euthanized.

### Real-time quantification PCR (qRT‒PCR) analysis

The mRNA level of genes in glioma cells was measured by qRT‒PCR using a Bio-Rad CFX96 Real-Time PCR System according to the standard protocol (Bio-Rad Laboratories, Inc., Hercules, CA, USA). Relative mRNA expression levels were calculated using the 2-ΔΔCt method. The primers are shown in Supplementary Table 1.

### Cell Culture

U251MG and U118MG cell lines were cultured in DMEM medium, supplemented with 10% fetal bovine serum (FBS) and 1% penicillin/streptomycin. The cells were maintained in a humidified incubator at 37 °C with 5% CO_2_.

### Cellular ROS measurement

Reactive oxygen species (ROS) production was measured by flow cytometry using redox-sensitive dyes (Invitrogen), including dihydroethidium (DHE), 2′,7′-dichlorofluorescein diacetate (H2DCFDA), MitoSOX Red and C11-BODIPY665/676, FlowJo (Tree Star) was used to analyzed median fluorescence in the relevant detection channels. ROS fold induction was calculated by dividing the median fluorescence of experimental samples by that of control samples.

### Cell viability assay

Tumor cells were seeded in 96-well plates at a density of 6 × 10^3^ cells/well. After adherence, cells were treated with ML162 (1 or 2 μM; #S4452; SelleckChem), RSL3 (1 or 2 μM; #19288, Cayman Chemicals), and ML210 (1, 3, or 6 μM; #S0788; SelleckChem), with or without Liproxstatin-1 (1 μM; #7699, SelleckChem) for 24 h. For the combination of liproxstatin-1 and ferroptosis activators, liproxstatin-1 was added 30 min before the activators. The cells were then incubated with 10 μL of Cell Counting Kit-8 (CCK8) solution in 100 μL of culture medium at 37 °C in a 5% CO_2_ incubator for 1 h. Subsequently, the 96-well plates were removed from the incubator, and the absorbance of the solution in each well was determined using a microplate reader at 450 nm. Cell viability was then calculated for each condition.

### Glutathione analysis

Intracellular GSH and GSSG levels were measured using the 5,5′-dithio-bis (2-nitrobenzoic acid)-glutathione reductase recycling method [23]. In brief, the rate of 5-thio-2-nitrobenzoic acid (TNB) formation was assessed by monitoring the change in absorbance at 412 nm. Concentrations of total and oxidized glutathione were calculated using linear regressions based on standard curves, and the reduced glutathione concentration was derived. Glutathione levels were normalized to protein concentration, which was measured using the BCA Kit (Pierce).

### Luciferase reporter assay

The promoter region of TP63, along with its two mutant forms (Mut1 and Mut2), was subcloned into the pGL4-luc vector (OBIO, Shanghai, China) containing the firefly luciferase reporter gene to generate three reporter constructs: TP63 WT, TP63 Mut1, and TP63 Mut2. The pRL-CMV vector, encoding Renilla luciferase, was co-transfected as an internal control. Dual-luciferase reporter assays were performed using the Dual-Luciferase® Reporter Assay System (Promega, Madison, WI, USA) following the manufacturer’s protocol.

### ChIP-qPCR

For the chromatin immunoprecipitation (ChIP) assay, cells were cross-linked and lysed, and chromatin was sheared and incubated with specific antibodies against β-catenin, ΔNp63, or EP300, or H3K27ac, or with IgG as a negative control. Immunoprecipitated DNA was purified and analyzed by quantitative real-time PCR (qRT-PCR) using specific primers targeting predicted binding regions. The sequences of the primers used for ChIP-qPCR are listed in the Supplementary Table 1.

### In vitro ubiquitination assay

To evaluate protein ubiquitination, cells were incubated with the proteasome inhibitor MG-132 for 10 h. After lysis, cell extracts were immunoprecipitated using an anti-β-catenin antibody. The precipitated proteins were then subjected to western blot analysis with a mouse anti-ubiquitin antibody to detect ubiquitinated β-catenin.

### Statistical analysis

The data were statistically analyzed by GraphPad Prism 8 software (Version 8.0.0) and R software (Version 4.1.0) and are presented as the mean ± standard deviation (S.D.) or standard error (S.E.M.). Differences were analyzed using Student’s *t* test for two groups and one-way ANOVA for multiple groups. A p value < 0.05 was considered statistically significant. *P < 0.05; **P < 0.01; ***P < 0.001; ****p < 0.0001.

## Supplementary information

SUPPLEMENTAL MATERIAL

WB uncropped

## Data Availability

The analysis data were accessed from TCGA database (https://portal.gdc.cancer.gov/), Further inquiries can be directed to contact the corresponding authors.
