# Supplementary material for: TP63 as a modulator of ferroptosis in TP53 mutations glioblastoma
Source: Cell Death Dis. 2025 Aug 13;16(1):614. doi: 10.1038/s41419-025-07938-w (PMC12343825; doi:10.1038/s41419-025-07938-w)
Supplement: Supplementary file 1 — SUPPLEMENTAL MATERIAL [file 41419_2025_7938_MOESM1_ESM.docx]

Supplementary table 1. Primers of the relative genes and siRNA sequence

| Gene | Sequence of primer (5’-3’) |
| --- | --- |
| TP63-F | GGACCAGCAGATTCAGAACGG |
| TP63-R | AGGACACGTCGAAACTGTGC |
| TAP63-F | CGTAGAAACCCCAGCTCATTTCT |
| TAP63-F | TGTTCCAGAAAATCCCAGATATGCTG |
| CTNNB1-F | AAAGCGGCTGTTAGTCACTGG |
| CTNNB1-R | CGAGTCATTGCATACTGTCCAT |
| CCND1-F | GCTGCGAAGTGGAAACCATC |
| CCND1-R | CCTCCTTCTGCACACATTTGAA |
| GPX4-F | GAGGCAAGACCGAAGTAAACTAC |
| GPX4-R | CCGAACTGGTTACACGGGAA |
| GCLC-F | GGAGGAAACCAAGCGCCAT |
| GCLC-R | CTTGACGGCGTGGTAGATGT |
| GSS-F | GGGAGCCTCTTGCAGGATAAA |
| GSS-R | GAATGGGGCATAGCTCACCAC |
| GSR-F | CACTTGCGTGAATGTTGGATG |
| GSR-R | TGGGATCACTCGTGAAGGCT |
| IDH2-F | CGCCACTATGCCGACAAAAG |
| IDH2-R | ACTGCCAGATAATACGGGTCA |
| GPX2-F | GGTAGATTTCAATACGTTCCGGG |
| GPX2-R | TGACAGTTCTCCTGATGTCCAAA |
| Si-CTNNB1#1 | AGGTGCTATCTGTCTGCTCTA |
| Si-CTNNB1#2 | CGCATGGAAGAAATAGTTGAA |
| Si-TP63#1 | CCTAGTCATTTGATTCGAGTA |
| Si-TP63#2 | GAGTGGAATGACTTCAACTTT |
| TP63 (P2) TBE2-F | GGAGTCCAGGTGGAAGTTGA |
| TP63 (P2) TBE2-R | CTTCTGGCTCCAGGATTTTG |
| GPX4 enhancer-F | CTGTGCTGGCTGCAGTGCTG |
| GPX4 enhancer-R | GGTGTGGGTGCCCAACCCAT |
| GAPDH promoter-F | TACTAGCGGTTTTACGGGCG |
| GAPDH promoter-R | TCGAACAGGAGCAGAGAGCGA |

Supplementary table 2. Antibody of the relative genes.

| Antibodies | Source | Identifier |
| --- | --- | --- |
| anti-p53 | Santa Cruz Biotechnology | Sc-126 |
| anti-GAPDH | Abcam | Ab181602 |
| HRP-conjugated Affinipure Goat Anti-Rabbit IgG(H+L) | Proteintech | Cat#SA00001-2 |
| HRP-conjugated Affinipure Goat Anti-Mouse IgG(H+L) | Proteintech | Cat#SA00001-1 |
| anti-β-catenin | Proteintech | Cat# 51067-2-AP |
| anti-p63 | Abcam | ab32353 |
| anti-cyclinD1 | Abcam | ab134175 |
| anti-GPX4 | Abcam | ab125066 |
| [Purified anti-p63 (TA) Antibody](https://www.biolegend.com/en-us/products/purified-anti-p63-ta-antibody-19799) | Biolegend | TAp63-4.1 |
| DeltaN p63 (E6Q3O) Rabbit mAb | Cell Signaling Technology | 6782S |
| p300 (D2X6N) Rabbit mAb | Cell Signaling Technology | 54062 |
| Acetyl-Histone H3 (Lys27) (D5E4) XP® Rabbit mAb | Cell Signaling Technology | 8173 |

Supplementary table 3. Reagents and plasmids.

| Antibodies | Source | Identifier |
| --- | --- | --- |
| ML162 | SelleckChem | #S4452 |
| ML210 | SelleckChem | #S0788 |
| WNT3a | Fisher Scientific | Cat# 5036WN010CF |
| IWR-1 | Santa Cruz | sc-295215A |
| RSL3 | Cayman Chemicals | #19288 |
| erastin | Cayman Chemicals | #17754s |
| liproxstatin-1 | SelleckChem | #7699 |
| MG-132 | Selleck Chemicals | S2619 |
| Doxycycline-inducible sgRNA lentiviral vectors targeting p53 | Addgene | 85535 |
| pCMV-TP53(human)-R273H-3×FLAG-Neo | MIAOLING Biology | P65314 |
| TP53-sgRNA#1 | iGeneBio | HTN330578 |
| TP53-sgRNA#2 | iGeneBio | HTN342005 |
| pEnCMV-FLAG-β-catenin(human)-SV40-Neo | MIAOLING Biology | P5614 |

**Supplementary Figure 1. TP53 mutations inhibit ferroptosis**


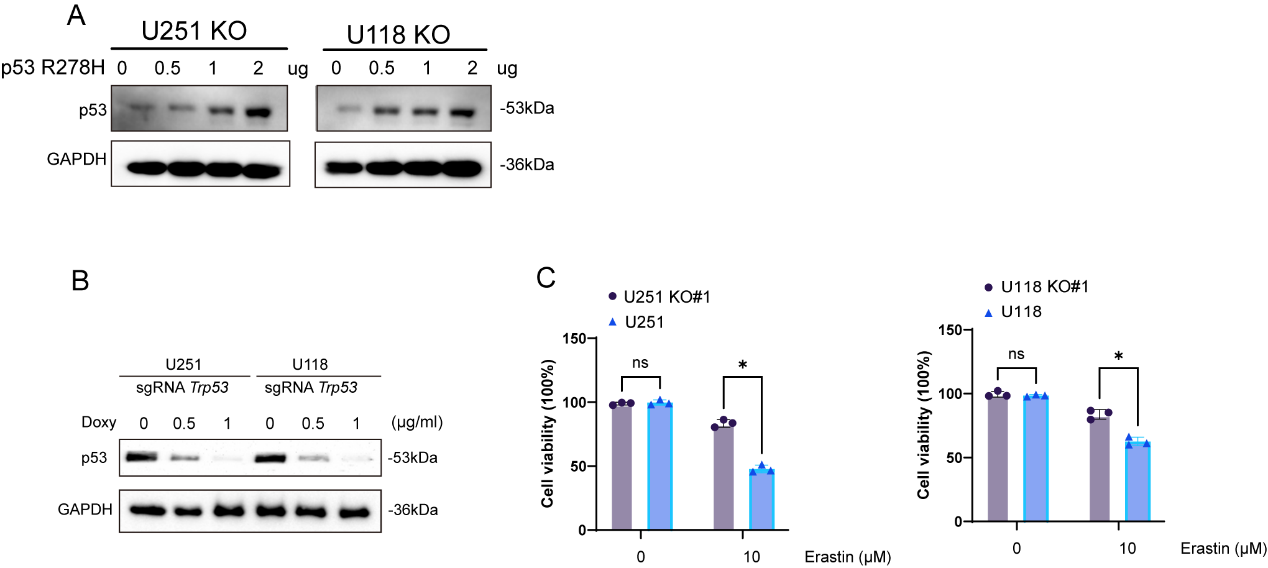


**A.** Western blot analysis of p53 expression in TP53 knockout (KO) cells transfected with varying amounts of the p53 R273H plasmid. **B.** Western blot analysis of p53 in the indicated cells, which express doxycycline-inducible plasmids targeting mutant p53, was performed with or without doxycycline treatment. **C.** The impact of Erastin on cells was evaluated 24 hours after treatment. Data are presented as mean ± SEM (n = 3). *p < 0.05, **p < 0.01, ***p < 0.001, ****p < 0.0001.

**Supplementary Figure 2. TP53 mutations upregulated** **ΔNp63 expression**


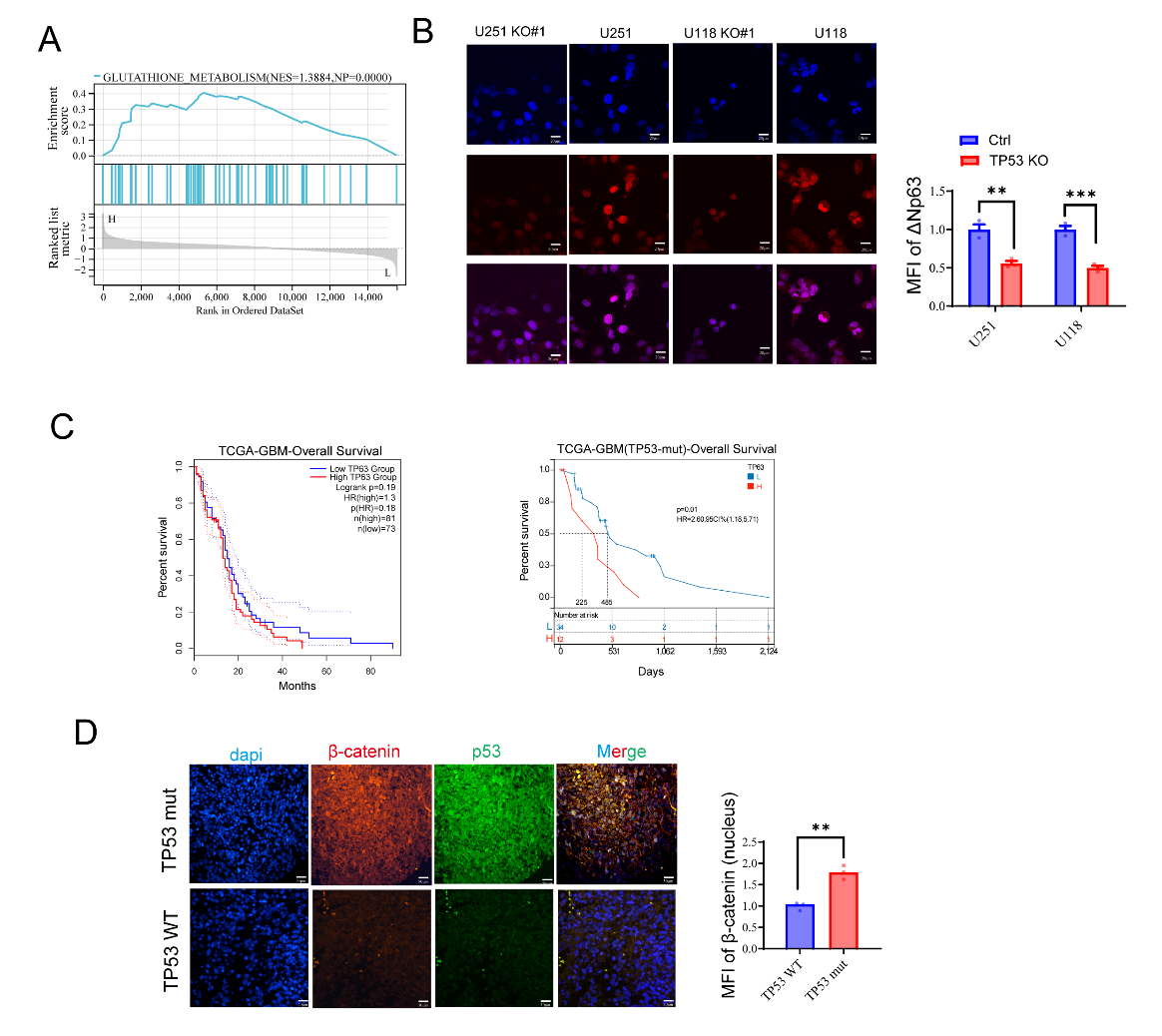


**A.** GSEA showing significant enrichment of glutathione metabolism pathways in the TP53 mutation relative gene set. **B.**  Immunofluorescence staining showing reduced ΔNp63 (red) in cells with or without TP53 KO. Nuclei (blue) were counterstained with DAPI. Scar bar = 20 μm. **C.** Kaplan–Meier survival analysis of GBM patients stratified by TP63 mRNA expression levels in the presence or absence of TP53 mutations. **D.** presentative immunofluorescence images of GBM tissue showing β-catenin (red), p53 (green), and nuclei (DAPI, blue) in tumors with or without TP53 mutations. Merged images indicate nuclear co-localization of β-catenin and p53. Scale bar, 20 μm. Right panel: Quantification of nuclear β-catenin mean fluorescence intensity (MFI). Data are presented as mean ± SEM (n = 3). *p < 0.05, **p < 0.01, ***p < 0.001, ****p < 0.0001.
