# Supplementary material for: TP63 as a modulator of ferroptosis in TP53 mutations glioblastoma
Source: Cell Death Dis. 2025 Aug 13;16(1):614. doi: 10.1038/s41419-025-07938-w (PMC12343825; doi:10.1038/s41419-025-07938-w)

Fig. 2B

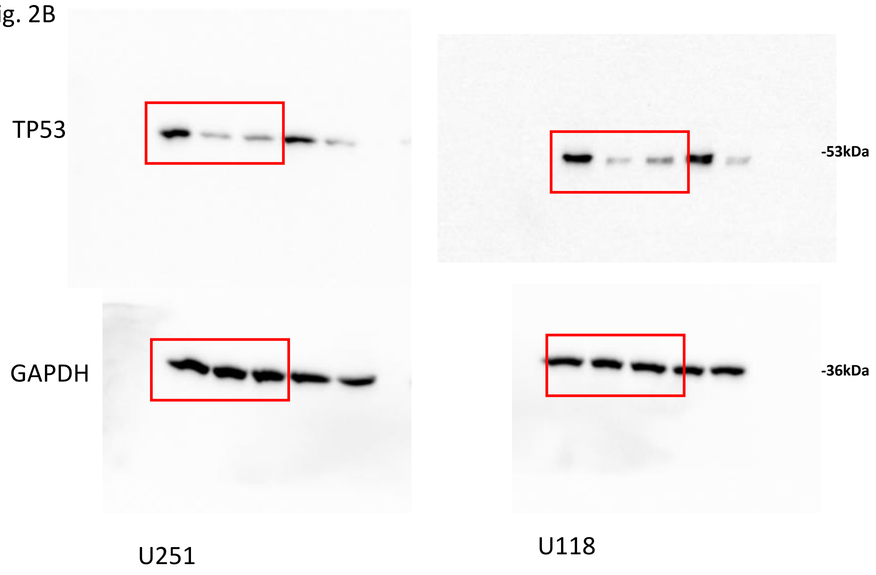

Fig. 3F

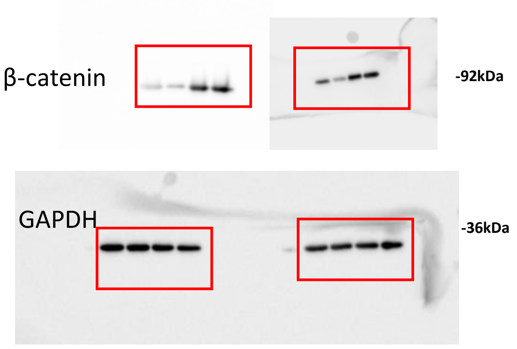

Fig. 3G

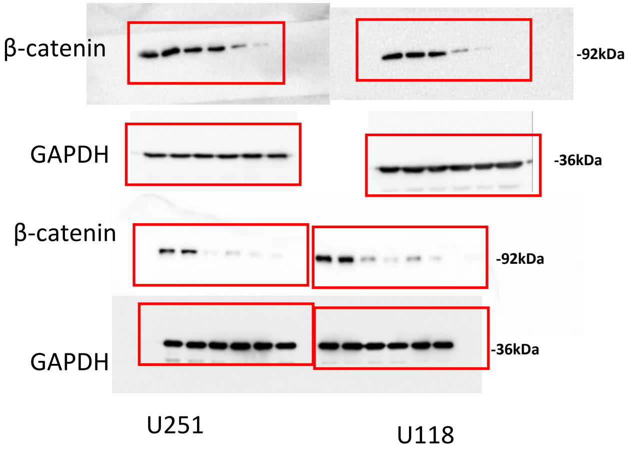

Fig. 3E

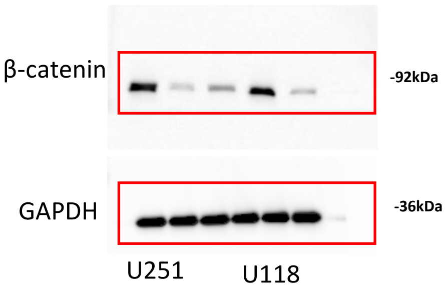

Fig. 3H

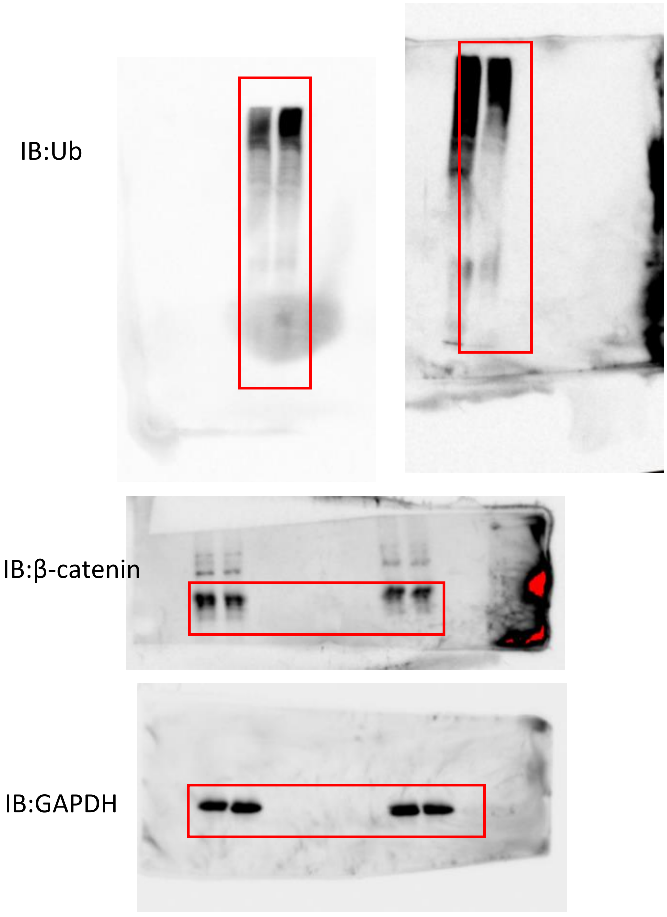

Fig. 4G

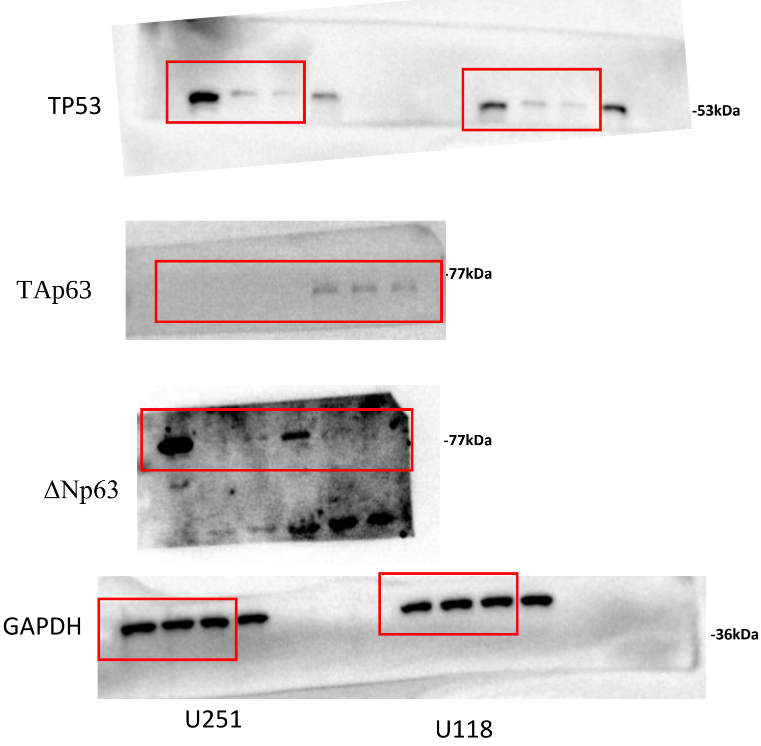

Fig. 4H

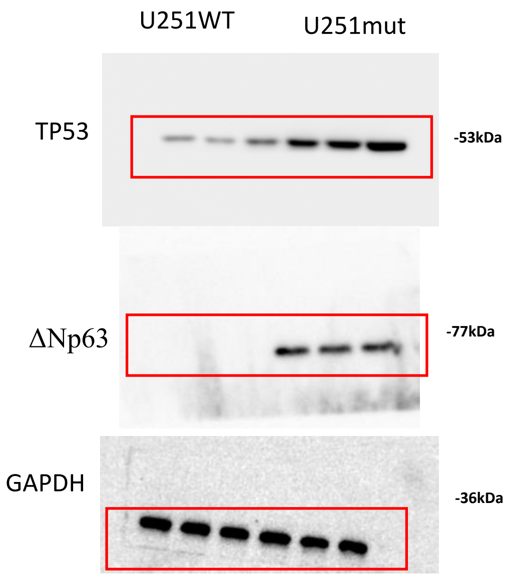

Fig. 4K

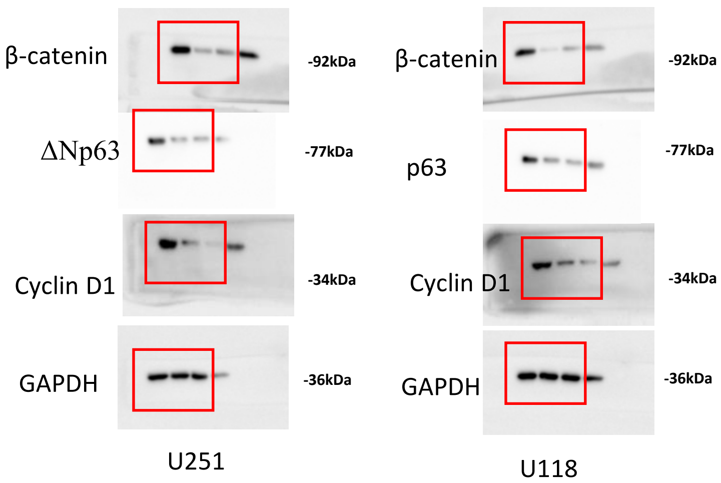

Fig. 4M

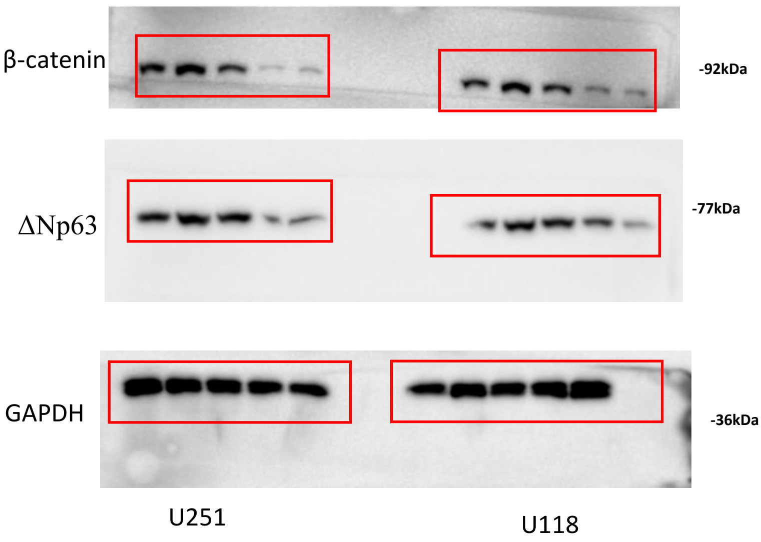

Fig. 5C

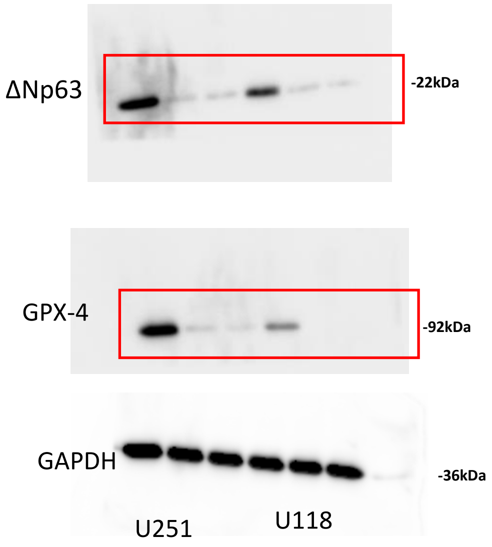

Fig. 5G

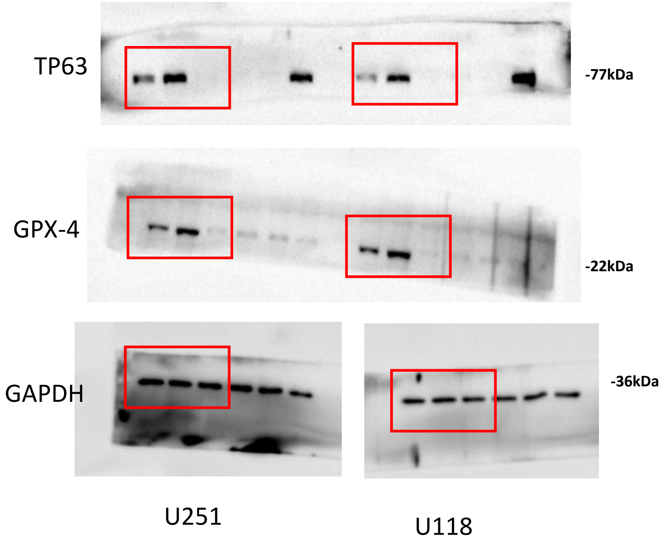

Fig. 5H

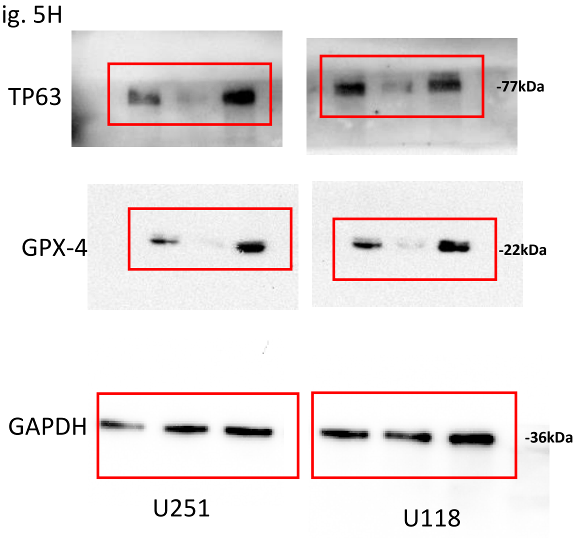

Fig. 5I

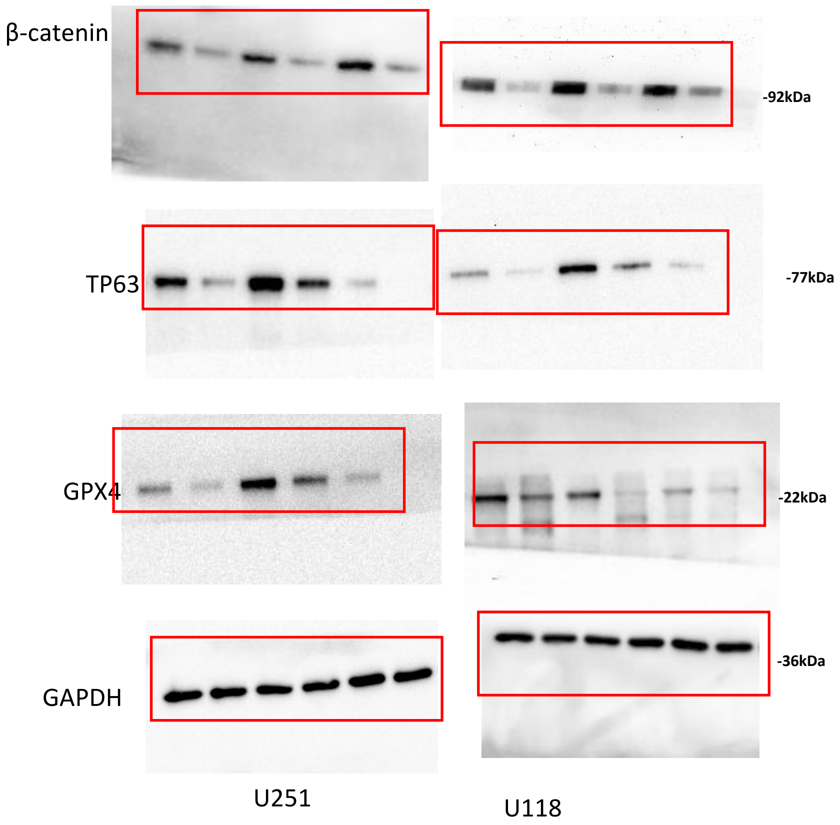

Fig. 6A

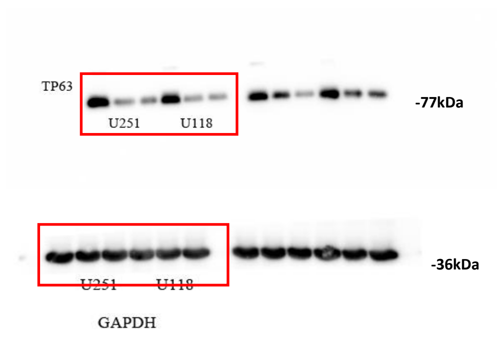

Supplementary figure 1A

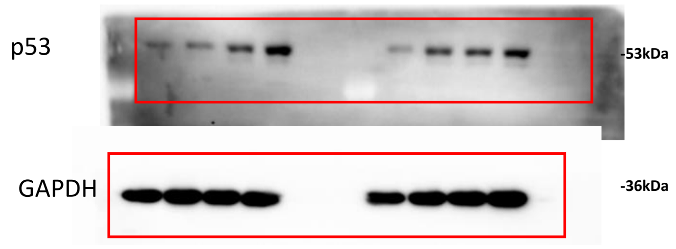

Supplementary figure 1B

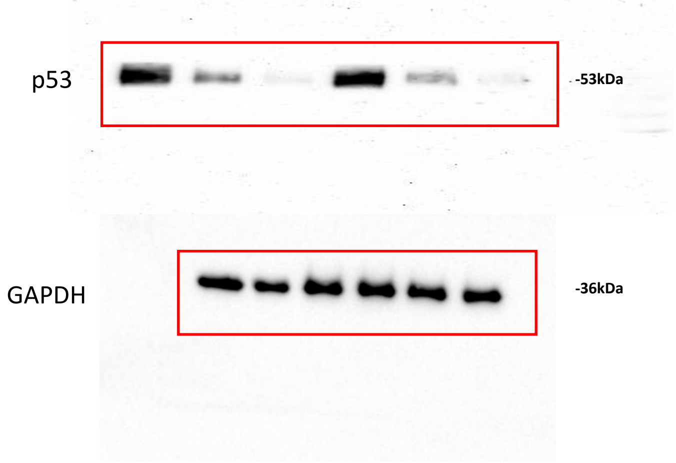

Supplement: Supplementary file 2 — WB uncropped [file 41419_2025_7938_MOESM2_ESM.pdf]
